# Prolactin Increases the Frequency of Follicular T Helper Cells with Enhanced IL21 Secretion and OX40 Expression in Lupus-Prone MRL/lpr Mice

**DOI:** 10.1155/2021/6630715

**Published:** 2021-03-08

**Authors:** Yolanda P. Alemán-García, Ricardo M. Vaquero-García, Rocio Flores-Fernández, Ezequiel M. Fuentes-Pananá, Patricia Gorocica-Rosete, Alberto Pizaña-Venegas, Luis Chávez-Sanchéz, Francico Blanco-Favela, María V. Legorreta-Haquet, Adriana K. Chávez-Rueda

**Affiliations:** ^1^UIM en Inmunología, Hospital de Pediatría, CMN Siglo XXI, Instituto Mexicano del Seguro Social, Mexico; ^2^Unidad de Investigación en Virología y Cáncer, Hospital Infantil de México “Federico Gómez”, Mexico; ^3^Departamento de Investigación en Bioquímica, Instituto Nacional de Enfermedades Respiratorias “Ismael Cosió Villegas”, Mexico; ^4^Unidad de Investigación y Bioterio, Instituto Nacional de Enfermedades Respiratorias “Ismael Cosió Villegas”, Mexico

## Abstract

Systemic lupus erythematosus is characterized by high levels of IgG class autoantibodies that contribute to the pathophysiology of the disease. The formation of these autoantibodies occurs in the germinal centers, where there is cooperation between follicular T helper cells (T_FH_) and autoreactive B cells. Prolactin has been reported to exacerbate the clinical manifestations of lupus by increasing autoantibody concentrations. The objective of this study was to characterize the participation of prolactin in the differentiation and activation of T_FH_ cells, by performing in vivo and in vitro tests with lupus-prone mice, using flow cytometry and real-time PCR. We found that T_FH_ cells express the long isoform of the prolactin receptor and promoted STAT3 phosphorylation. Receptor expression was higher in MRL/lpr mice and correlative with the manifestations of the disease. Although prolactin does not intervene in the differentiation of T_FH_ cells, it does favor their activation by increasing the percentage of T_FH_ OX40^+^ and T_FH_ IL21^+^ cells, as well as leading to high serum concentrations of IL21. These results support a mechanism in which prolactin participates in the emergence of lupus by inducing overactive T_FH_ cells and perhaps promoting dysfunctional germinal centers.

## 1. Introduction

The neuroendocrine and immune systems are closely interrelated, as the secretory products of the neuroendocrine system can act on the immune system and vice versa [1]. One example involves hormones that can regulate the immune system [2, 3], such as prolactin (PRL) secreted by the pituitary gland, and extrapituitary immune system cells, such as T cells [4, 5], B cells, antigen presenting cells (APCs) [6], natural killer cells [7, 8], and monocytes/macrophages [9]. The immunostimulatory functions of PRL have been previously described. PRL favors the differentiation of thymocytes [10], increasing the expression of CD69 and CD25 in activated CD8^+^ T cells [11]. In CD4^+^ T cells, autocrine PRL is important for maintaining the expression of CD69 and CD40L and the secretion of IL2 and IFN-*γ* [5]. In a CD4^+^ T cell line, PRL induced T-bet transcription through phosphorylation of JAK2 and STAT5 [12]. In addition, hyperprolactinemia has been detected in many patients with different autoimmune diseases [13–15], including systemic lupus erythematosus (SLE), where it has been associated with disease activity [16, 17], with the concentration of anti-dsDNA antibodies [18], anemia, and all types of serositis [19]. SLE is a chronic autoimmune disease characterized by the presence of autoantibodies targeting DNA, RNA, histones, RNP, Ro, La, etc. [20]. These antibodies are from the IgG isotope, which form immune complexes that are deposited in any organ, causing damage. The prevalence of SLE is approximately ninefolds higher in women than in men, and it increases after puberty and decreases after menopause [21]. There are well-established experimental models mimicking many aspects of SLE, such as the MRL/lpr mouse strain [22]. Raising serum PRL levels in this strain, we demonstrated that the concentration of IgG isotype anti-dsDNA autoantibodies increased, resulting in earlier and more severe manifestations of the disease [23, 24].

In the different mouse models that develop SLE, there is an increase in the spontaneous formation of germinal centers (GCs), which correlates with the beginning of the production of autoantibodies [25, 26]. GCs provide a proper microenvironment for the activation, somatic diversification, and affinity maturation of autoreactive B cells, which occur before the production of autoantibodies [27, 28]. GC formation depends on the presence of follicular T helper cells (T_FH_), a specialized subpopulation of CD4 T cells. T_FH_ cells are characterized by their expression of CXCR5, ICOS, PD1, CD154, and transcription factor BCL6, in addition to secreting IL21 [29–33]. An increase in the frequency of circulating T_FH_ is reported in patients with SLE, having a positive correlation with autoantibody titers and disease activity [34–37]. Meanwhile, it has been observed that the clinical manifestations of the disease decrease upon inhibiting the expression of the IL21 receptor in mouse models [38]. Therefore, dysregulation of the T_FH_ response contributes to the production of pathogenic autoantibodies and, therefore, to the promotion of autoimmune diseases mediated by autoantibodies such as SLE [39].

Taking into account all aforementioned findings, we designed this study to determine the contribution that PRL has to the differentiation and activation of T_FH_ cells in the MRL/lpr mice. We found that T_FH_ cells express the long isoform of the PRL receptor and promoted STAT3 phosphorylation. Furthermore, PRL favors the dysregulation of T_FH_ cells by increasing both their absolute number and their activation.

## 2. Materials and Methods

### 2.1. Mice

All studies were approved by the Animal Care Committee of the Instituto Nacional de Enfermedades Respiratorias “Ismael Cosio Villegas” and the Hospital de Pediatría, Centro Medico Nacional Siglo XXI, IMSS (protocol numbers R-2016-785-050 and R-2017-785-114), and all mouse measurements were in accordance with the approved guidelines established by Mexico (Norma Oficial Mexicana NOM-062-ZOO-1999) and the NIH Guide for the Care and Use of Laboratory Animals. MRL/MpJFASlpr (MRL/lpr) mice were purchased from the Jackson Laboratory (Maine, USA), and C57BL/6 mice were purchased from the Instituto Nacional de Ciencias Médicas y Nutrición (CDMX Mexico). Mice were housed in a pathogen-free barrier facility and were provided with sterile food and water ad libitum.

### 2.2. Prolactin Hormone

We used murine recombinant PRL (National Hormone and Peptide Program, NIH).

### 2.3. Antibodies

All cells were labeled with the viability dye Ghost Red (Tonbo Bioscience, USA). The antibodies used for cell culture were as follows: anti-CD3 (clone 145-2C11) and anti-CD28 (clone 37.51) from Invitrogen, USA; anti-IFN-*γ* (clone XMG12) and anti-IL4 (clone 11B11) from BioLegend, USA; anti-TGF*β* from Peprotech, USA; cytokines IL6 and IL21 from Miltenyi Biotec, Germany. The antibodies used for staining were as follows: anti-mouse PRL receptor APC (clone T6, Novus Biologicals, USA); anti-CD4 PECy5 (clone GK1.5), anti-Ki-67 Alexa 488 (clone 16A8), anti-IL21 biotin (clone 7H20-I19-M3), and PE-conjugated streptavidin from BioLegend, USA; anti-CD44 PECy5 (clone IM7), anti-CD62L PE, (clone MEL-14), anti-BCL6 PE (clone BCL-DWN), anti-CXCR5 PECy7 (clone SPRCL5), and anti-PD1 APC (clone J43) from eBioscience, Invitrogen, USA; anti-ICOS VioGreen (clone 7E.17G9), anti-OX40 PE (clone REA625), and anti-AKT PE (clone REA677) from Miltenyi Biotec, Germany; and anti-STAT1 PE (clone A15158B), anti-STAT3 PE (clone 13A3-1), and anti-STAT5 PE (clone SRBC2X) from BioLegend.

### 2.4. Induction of High Prolactin Levels and Assessment of SLE Manifestations

MRL/lpr and C57BL/6 female mice (9-weeks-old) were subcutaneously injected with (i) 200 *μ*g of metoclopramide (Sigma-Aldrich, US) in 100 *μ*L of PBS, (ii) 0.6 mg/kg of bromocriptine (Santa Cruz Biotechnology, USA) in 100 *μ*L of PBS, (iii) 100 *μ*L of PBS, or (iv) no treatment for 6 weeks. Urinary protein levels were assessed semiquantitatively using reagent strips for urinalysis (Mission, USA). Serum samples obtained at the beginning and at the end of the experiments were kept at −35°C until they were assayed for anti-dsDNA antibodies as we have previously reported [23, 24].

### 2.5. Serum IL21 Concentration

For the detection of IL21 in sera, the commercial Legend Max Mouse IL21 ELISA kit (BioLegend, USA) was used according to the supplier's instructions. For each determination, 50 *μ*L of serum was used. The plate was read in the ELISA reader (Dynatech MR5000) at 450 nm.

### 2.6. Purification of T_naïve_ and T_FH_ Cells from the Spleen

Eighteen-week-old mice were euthanized, and spleen cells were collected with cold RPMI supplemented with 2% FBS and 2 mM EDTA (IBI Scientific, USA). Red blood cells were depleted with lysis buffer (Sigma-Aldrich, USA) and incubated with anti-CD4 MicroBeads (for T CD4 cells, Miltenyi Biotec); they were selected with the magnetically activated cell sorting (MACS) system (Miltenyi Biotec, Germany) through positive selection using LS columns (Miltenyi Biotec). Single-cell suspensions of CD4^+^ T cells were incubated with fluorescently labeled antibodies specific for CD44, CD62L, CXCR5, and PD1 in staining buffer (PBS with 0.5% BSA) for 20 min at 4°C. Further, the cells were incubated with DAPI to select living cells (DAPI^−^), washed, and T_naïve_ (CD44^−^CD62L^+^) and T_FH_ cells (CXCR5^+^PD1^+^) were isolated. Cell sorting was performed using a FACS Influx Sorter (BD Biosciences). The purity of sorted cells ranged from 95% to 98%.

### 2.7. RT-PCR for Prolactin Receptor Isoforms

To determine the expression of PRL receptor isoforms, T_naïve_ and T_FH_ cells from 18-week-old MRL/lpr mice were purified by sorting with a BD Influx Cytometer. Real-time PCR was performed using the following primers synthesized by Integrated DNA Technologies (IDT, USA): *β*-actin (housekeeping control) 5′–GAGGAGGCTCTGGTTCAACA–3′ (left) and 5′–CAGTAAATGCCACGAACGAA–3′ (right). To determine the PRL receptor isoforms, three primers were used: common 5′–AAGCCAGACCATGGATACTGGAG–3′ (left), long isoform 5′–AGCAGTTCTTCAGACTTGCCCTT–3′ (right), and short isoform 5′–TTGTATTTGCTTGCAGAGCCAGT–3′ (right). The samples were run in the LightCycler II thermal cycler (Roche, Germany) under the following conditions: one cycle at 95°C for 15 min, 40 cycles at 95°C for 10 s, 61°C for 30 s, and 72°C for 30 s, and one cycle at 72°C for 30 s. The relative expression was analyzed using the 2^−*ΔΔ*Ct^ method. The murine breast cancer cell line EpH4 1424 was used as a positive control for the expression of the long and short PRL receptor isoforms.

### 2.8. Prolactin Receptor Expression (Protein)

CD4^+^ T cells from 9- and 18-week-old mice were isolated from the spleen with the CD4^+^ T Cell Isolation Kit (Miltenyi Biotec, Germany) and stained with anti-mouse PRL receptor, anti-CD4, anti-CD44, and anti-CD62L for naïve T cells or anti-CD4, anti-CXCR5, and anti-PD1 T_FH_ cells.

### 2.9. Purification of T_naïve_

Nine-week-old mice were euthanized and spleen cells were collected with cold RPMI, and blood cells were depleted with lysis buffer. Naïve T cells were isolated from the spleen using a CD4^+^ naïve T cell (T_naïve_) Isolation Kit (BioLegend, USA), following the manufacturer's instructions.

### 2.10. T_FH_ Differentiation

T_naïve_ cells were differentiated to T_FH_ cells in the presence of the following antibodies and cytokines: anti-CD3, 2.5 *μ*g/mL; anti-CD28, 5 *μ*g/mL; anti-IFN-*γ*, 10 *μ*g/mL; anti-IL4, 10 *μ*g/mL; anti-TGF*β*, 20 *μ*g/mL; IL6, 10 ng/mL; IL21, 10 ng/mL; and with or without 50 ng/mL of PRL for 48 h at 37°C and 5% CO_2_.

### 2.11. Flow Cytometry of In Vitro Differentiated T_FH_ Cells

For OX40 expression, differentiated T_FH_ cells in vitro or splenocytes from mice that underwent different treatments were stained with Ghost Red (viability), anti-CD4, anti-CXCR5, anti-PD1, and anti-OX40. For intracellular IL21, cells were incubated with 1x Cell Stimulation cocktail (Invitrogen, USA) and 1x Protein Transport Inhibitor cocktail (Invitrogen, USA) for 5 h at 37°C and 5% CO_2_. Cells were stained with Ghost Red, as well as anti-CD4, anti-CXCR5, and anti-PD1 antibodies. To stain for intracellular proteins (BCL/6, Ki-67, and IL21), cells were fixed and permeated using a Foxp3/transcription factor staining buffer set (eBioscience, USA) or an Intracellular Fixation and Permabilization Buffer Set (eBioscience, USA) for the latter two. All FACS data were acquired with an MACSQuant Analyzer 10 flow cytometer (Miltenyi Biotec, Germany) and analyzed using the FlowJo software (Tree Star, USA).

### 2.12. Analysis of STATs and AKT Phosphorylation

T_FH_ cells differentiated in vitro were left to rest for 8 h in medium, then T_FH_ and T_naïve_ cells were incubated with PRL (50 ng/mL) for 30 min and fixed with 1x BD Phosflow Lyse/5x FIx Buffer (BD Biosciences, USA) for 10 min. Cells were permeabilized with Perm Buffer III from BD Phosflow (BD Biosciences) to determine STAT3, STAT1, STAT5, and AKT phosphorylation. Cells were washed with FACS buffer and incubated at 4°C for 30 min with the antibodies for flow cytometry analysis. Data were acquired using an MACSQuant Analyzer 10 cytometer (Miltenyi Biotec) and analyzed with FlowJo software (Tree Star, USA). To confirm STAT3 activation, T_FH_ and T_naïve_ cells were preincubated for 30 min in basal medium alone or with 10 mM of the STAT3 inhibitor (Stattic, Cell Signaling Technology, USA).

### 2.13. Statistical Analysis

The Shapiro–Wilk normality test was used to determine the distribution of data. The results were expressed as the mean and standard deviation. Differences between groups were determined using the ANOVA test. A *p* value < 0.05 was considered significant; statistical analysis of the data was performed using the SPSS Statistics 27 software.

## 3. Results

### 3.1. T_FH_ Cells Increase in Mice That Develop Lupus

**Figure 1:**
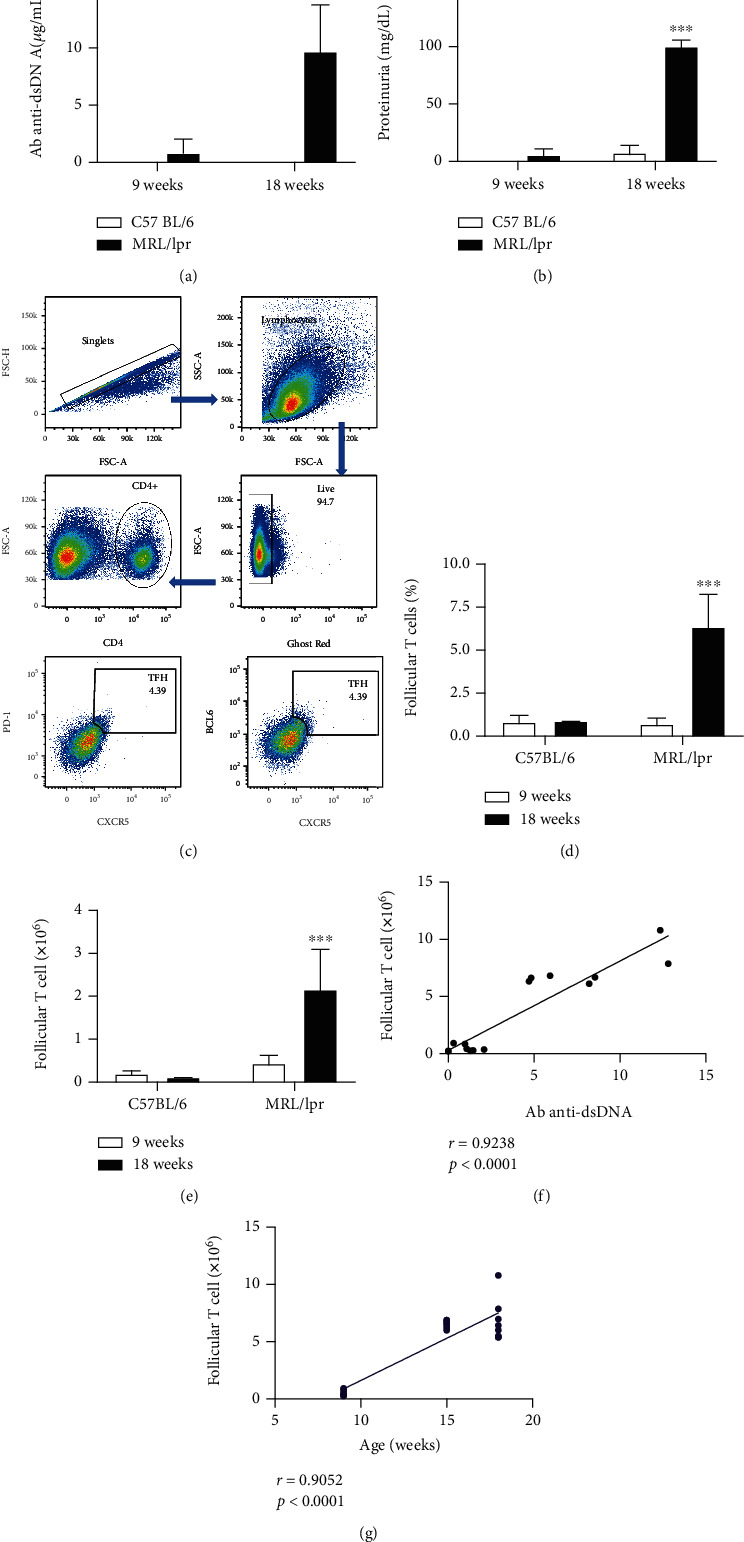
T_FH_ cells increase in mice that develop lupus. In 9- and 18-week-old mice of the C57BL/6 and MRL/lpr strains, the following were determined: (a) concentration of anti-dsDNA antibodies by ELISA and (b) levels of proteinuria using a test strip. (c) Demonstration of the gating strategy for the flow cytometry analysis of T_FH_ cells. Doublets were excluded by gating on FSC-H×FSC-A lymphocytes which were identified on the basis of their scatter properties (FSC-A×SSC-A plot), and live cells were gated in the Ghost Red^−^. The gate of CD4^+^ T cells was selected. The CXCR5^+^PD1^+^ or CXCR5^+^BCL6^+^ population represents T_FH_ cells. (d) Percentage and (e) absolute number of T_FH_ cells. Each determination was made in eight mice. Pooled data are presented as the mean ± SD; ^∗∗∗^*p* < 0.001 using ANOVA. Pearson's correlation between absolute number of T_FH_ cells and (f) anti-dsDNA antibody concentration and (g) age.

Murine models of SLE spontaneously increase the formation of GC, which, however, has not been further explored. Seeking for an explanation for this observation and given that the increased GC formation correlates with prodromal SLE features, we measured the percentage of T_FH_ cells in splenocytes of 9- and 18-week-old mice, in which the disease activity was determined by measuring the concentration of anti-dsDNA antibodies (IgG) and proteinuria. The 18-week-old MRL/lpr mice showed significantly elevated serum concentrations of anti-dsDNA antibodies (9.7 ± 3.97 *μ*g/mL) and proteinuria (100 ± 7.56 mg/dL) compared with 9-week-old mice (anti-dsDNA 0.82 ± 1.18 *μ*g/mL; proteinuria 4.29 ± 6.50 mg/dL). We did not or we barely detected anti-dsDNA antibodies and proteinuria in the control strain (C57BL/6) (Figures 1(a) and 1(b)).

We found that the 18-week-old MRL/lpr mice had a significantly higher percentage of T_FH_ (6.35% ± 1.98%) compared with the 9-week-old MRL/lpr mice (0.71% ± 0.43%) and C57BL/6 mice (9 weeks old, 1.22% ± 0.90%; 18 weeks old, 0.88% ± 0.08%) (Figures 1(c) and 1(d)). A similar behavior was observed with the cell absolute numbers; 18-week-old MRL/lpr mice had a higher number of T_FH_ cells (2.16 ± 0.97 × 10^6^ cells/spleen) compared with 9-week-old MRL/lpr mice (0.40 ± 0.24 × 10^6^ cells/spleen) and C57BL/6 mice (9 weeks old, 0.26 ± 0.19 × 10^6^ cells/spleen; 18 weeks old, 0.11 ± 0.01 × 10^6^ cells/spleen) (Figure 1(e)). Therefore, in lupus-prone MRL/lpr mice, the increased formation of GCs may be at least partially explained by the increased formation of T_FH_ cells. Indeed, T_FH_ cell numbers correlated with autoantibody concentrations and with age (Figures 1(f) and 1(g)).

### 3.2. T_naïve_ and T_FH_ Cells Express the Long Isoform of the PRL Receptor

**Figure 2:**
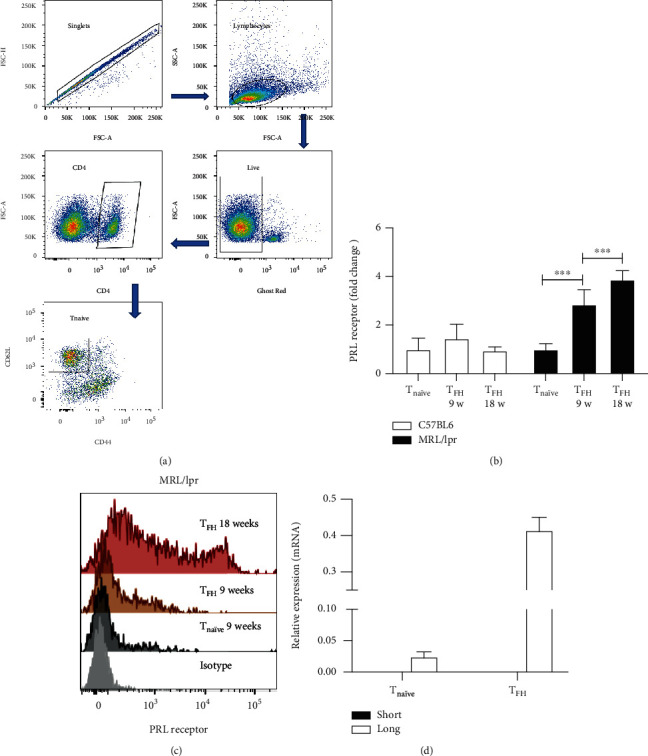
PRL receptor expression on T_naïve_ and T_FH_ cells. Splenocytes from 9- and 18-week-old C57BL/6 and MRL/lpr mice were stained with anti-CD4, anti-CXCR5, and anti-PD1 for T_FH_ cells (as shown in Figure 1(c)) and with anti-CD4, anti-CD44, and anti-CD62L for T_naïve_ cells, then cells were stained with an anti-PRL receptor antibody. (a) Demonstration of the gating strategy for the flow cytometry analysis of T_naïve_ cells. Doublets were excluded by gating on FSC-H×FSC-A, lymphocytes were identified on the basis of their scatter properties (FSC-A×SSC-A plot), and live cells were gated in the Ghost Red^−^. The gate of CD4^+^ T_naïve_ was selected (CD62L^+^ CD44^−^). (b) Expression of the PRL receptor is reported as the fold change in receptor expression with respect to PRL receptor expression in T_naïve_ cells. (c) Representative histogram of PRL receptor expression in MRL/lpr mouse cells. The measurement was carried out in duplicate in six mice per group. Pooled data are presented as the mean ± SD; ^∗∗∗^*p* < 0.001 using ANOVA. (d) T_naïve_ and T_FH_ cells from 18-week-old MRL/lpr mice were purified by Sort, and the isoform of the PRL receptor was determined by real-time (RT-) PCR. The murine breast cancer cell line EpH4 1424 was used as a positive control for the expression of the long and short PRL receptor isoforms (not shown). Two different experiments were performed; in each experiment, a pool of cells isolated from three mice was used.

To explore whether the formation of T_FH_ cells may be influenced by PRL, we determined the expression pattern of the PRL receptor between lupus-prone and control mice, reporting the expression of the PRL receptor as the fold change in T_FH_ cells with respect to that of 9-week-old T_naïve_ cells. We did not find an increase in the PRL receptor expression in T_FH_ cells of 9- and 18-week-old C57BL/6 mice. On the contrary, the MRL/lpr strain showed augmented expression, both at 9 (2.85 ± 0.56-fold change) and at 18 weeks of age (3.87 ± 0.33-fold change), with T_FH_ cells of the 18-week-old mice exhibiting the greatest expression (Figures 2(a)–2(c)); we have previously made a similar observation in B cell splenocytes [24]. We observed that both T_naïve_ and T_FH_ cells of MRL/lpr mice only express the long isoform of the PRL receptor (Figure 2(d)).

### 3.3. Prolactin Increases the Absolute Number of T_FH_ OX40^+^ Cells and IL21-Secreting Cells

**Figure 3:**
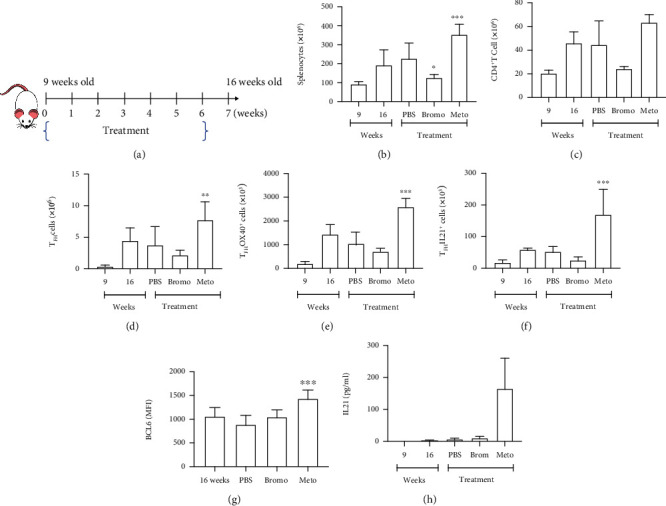
Metoclopramide increases the absolute number of T_FH_ populations in MRL/lpr mice. Nine-week-old MRL/lpr mice were treated with metoclopramide (meto), bromocriptine (bromo), or PBS or were left without intervention (left column marked by age in weeks) for 6 weeks. (a) Flow chart of treatment strategy. At the end of the treatment, cells were labeled with anti-CD4, anti-CXCR5, anti-PD1, anti-OX40, anti-BCL6, or anti-IL21 antibodies. The graphs show the absolute number of (b) splenocytes, (c) CD4^+^ T cells, (d) T_FH_ cells, (e) OX40^+^ T_FH_ cells, and (f) IL21^+^ T_FH_ cells. (g) Expression of BCL6 in T_FH_ cells. (h) IL21 concentration in serum. For the determination of T_FH_ IL21^+^, the cells were stimulated with stimulation cocktail (PMA ionomycin) for 5 h and then stained. MFI: mean fluorescent intensity. Eight mice per condition were used. Pooled data are presented as the mean ± SD; ^∗∗∗^*p* < 0.001, ^∗∗^*p* < 0.01, and ^∗^*p* < 0.05 using ANOVA.

We previously reported in MRL/lpr mice that pharmacologically raising serum PRL levels with metoclopramide exacerbates the clinical manifestations of SLE, with an increase in autoantibody concentration, as well as proteinuria [23, 24]. To determine whether PRL could affect the number of T_FH_ cells, as well as their activation in vivo, we treated MRL/lpr mice with metoclopramide (to increase PRL levels), bromocriptine (to decrease PRL levels), or PBS (Figure 3(a)). We found that the absolute number of splenocytes spontaneously increased with age, as it was observed even in MRL/lpr mice treated with PBS (16 weeks 235.36 ± 78.21 × 10^6^ cells). Still, this increase was more significant in mice treated with metoclopramide (348.84 ± 52.71 × 10^6^ cells), while the numbers of splenocytes in the bromocriptine condition (138.80 ± 25.95 × 10^6^ cells) were closer to the 9-week baseline (92.27 ± 12.45 × 10^6^ cells) (Figure 3(b)). A similar observation was made for the absolute numbers of CD4^+^ T cells and T_FH_ cells, as well as for activated T_FH_ OX40^+^ cells and T_FH_ IL21^+^ cells. For all these populations, the highest absolute numbers were from mice treated with metoclopramide and the lowest for the bromocriptine condition. CD4^+^ T cells are composed of the following: metoclopramide 63.58 ± 6.15 × 10^6^ cells, PBS 44.74 ± 18.71 × 10^6^ cells, and bromocriptine 24.57 ± 1.22 × 10^6^ cells (Figure 3(c)). T_FH_ populations are composed of the following: metoclopramide (T_FH_7.69 ± 2.66; T_FH_ OX40^+^2.35 ± 0.60; T_FH_ IL21^+^0.14 ± 0.07 × 10^6^ cells), PBS (T_FH_3.77 ± 2.72; T_FH_ OX40^+^1.03 ± 0.43; T_FH_ IL21^+^0.05 ± 0.01 × 10^6^ cells), and bromocriptine (T_FH_2.20 ± 0.59; T_FH_ OX40^+^0.70 ± 0.11; T_FH_ IL21^+^0.03 ± 0.01 × 10^6^ cells) (Figures 3(d)–3(f)). Therefore, the increased number of splenocytes observed in each condition mirrors the numbers of each of these CD4 populations that participate in GC formation. We did not observe differences in the absolute numbers of these cells between PBS treated or untreated MRL/lpr mice.

We determined the expression of BCL6 in T_FH_ cells, observing an increase only in mice treated with metoclopramide (Figure 3(g)). Furthermore, the serum levels of IL21 were also more elevated in mice treated with metoclopramide (Figure 3(h)). Meanwhile, we did not observe changes in the numbers of these populations in C57BL/6 mice (Figure S1, Supplementary Materials).

### 3.4. Prolactin Does Not Affect the Survival or Differentiation of T_FH_ Cells

**Figure 4:**
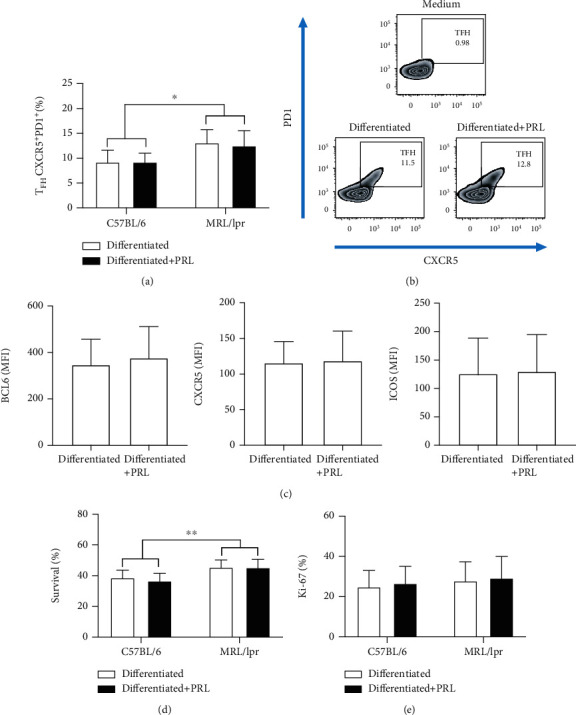
Differentiation and survival of T_FH_ cells in the presence of PRL. T_naïve_ cells from 9-week-old C57BL/6 and MRL/lpr mice were purified by MACS and differentiated to T_FH_ in the presence and absence of PRL for 48 h, before staining with a viability marker (Ghost Red) and anti-Ki-67, anti-CD4, anti-CXCR5, anti-BCL6, and anti-ICOS antibodies. The surface CXCR5^+^PD1^+^ population represents T_FH_ cells. (a) Percentage of differentiation to T_FH_ in vitro. (b) Zebra plot of one representative experiment. (c) Expression of BCL6, CXCR5, and ICOS (MFI) in T_FH_ cells. (d) Percentage of T_FH_ cell survival. (e) Percentage of proliferation (Ki-67^+^). Six different experiments were performed; each experiment was done in triplicate. Pooled data are presented as the mean ± SD; ^∗∗^*p* < 0.01 and ^∗^*p* < 0.05 using ANOVA.

PRL has been reported to increase survival in immature B cells of mice that develop SLE [40]. We determined whether PRL could favor the survival and/or differentiation of T_FH_ cells as a mechanism to explain their increased numbers. For this, we isolated CD4 T_naïve_ cells from 9-week-old C57BL/6 and MRL/lpr mice and induced T_FH_ differentiation in culture. We did not find differences in the percentage of T_FH_ cells differentiated without PRL (C57BL/6 9.26% ± 3.50%; MRL/LPR 13.12% ± 3.26%) and with PRL (C57/BL6 8.56% ± 2.35%; MRL/lpr 12.50% ± 4.41%) (Figures 4(a) and 4(b)) nor did we find a difference in the expression (mean fluorescence intensity, MFI) of BCL6, ICOS, and CXCR5 (Figure 4(c)); although a greater differentiation to T_FH_ was observed in cells from MRL/lpr mice compared with cells from C57BL/6 mice. When we determined the survival of T_FH_ cells after the differentiation assay, we did not find significant differences between the percentage of live differentiated T_FH_ cells without PRL (C57BL/6 38.69% ± 4.83%; MRL/lpr 45.49% ± 4.72%) and with PRL (C57BL/6 36.62% ± 4.87%; MRL/lpr 45.33% ± 5.33%) (Figure 4(d)). Additionally, the cells differentiated to T_FH_ from MRL/lpr mice presented slightly better survival than those from C57BL/6 mice. We did not observe differences in the percentages of proliferating cells (Figure 4(e)).

### 3.5. Prolactin Activates T_FH_ Cells

**Figure 5:**
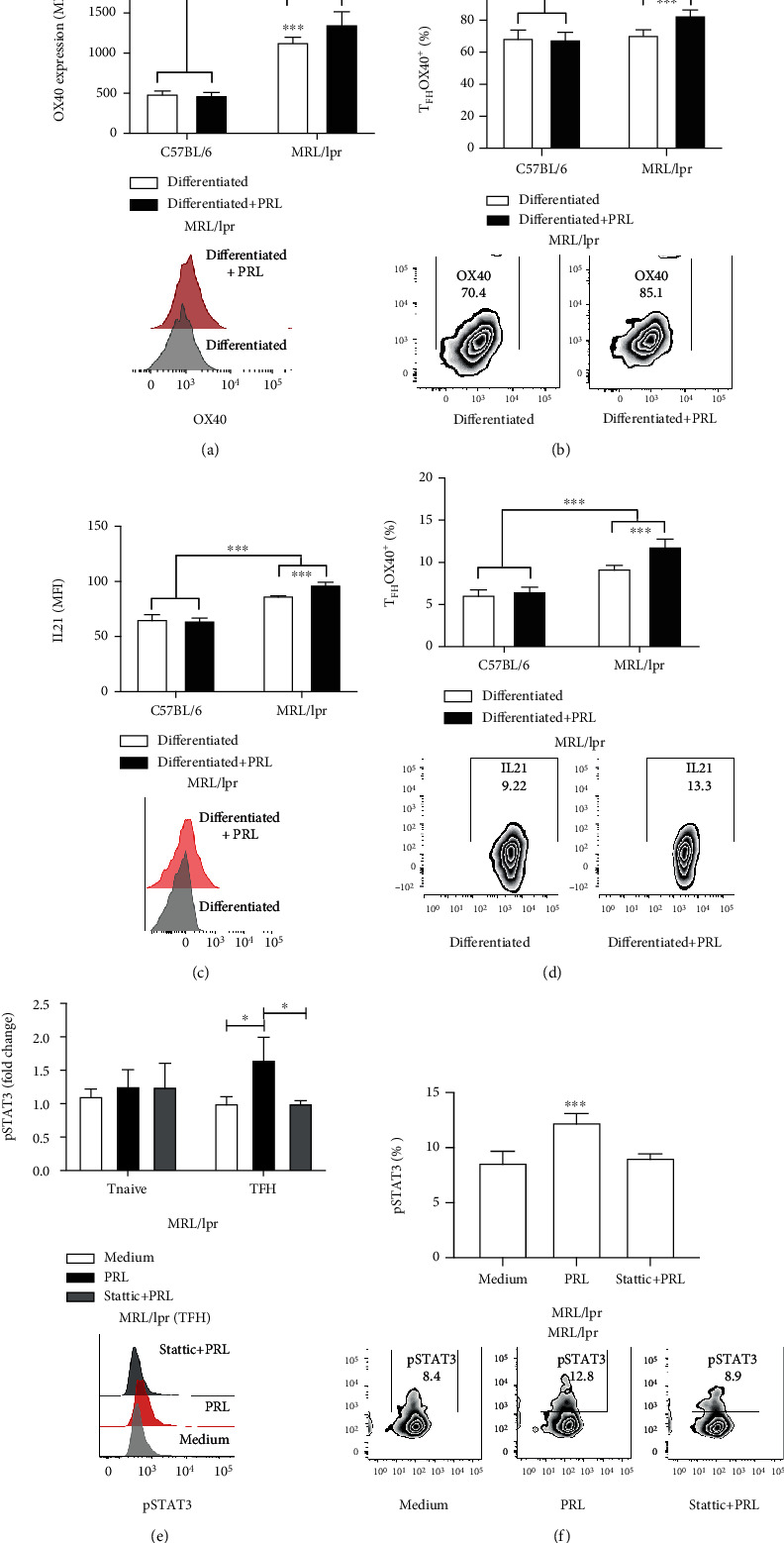
Activation and signaling of differentiated T_FH_ cells in vitro in the presence of PRL. T_naïve_ cells from 9-week-old C57BL/6 and MRL/lpr mice were purified by MACS and differentiated to T_FH_ with or without PRL. (a) Expression of OX40 (MFI) and representative histograms of OX40 expression in T_FH_ cells from MRL/lpr mice. (b) Percentage and representative Zebra plots of T_FH_ OX40^+^ cells. (c) Expression of IL21 (MFI) and representative histogram of IL21 expression in T_FH_ cells from MRL/lpr mice. (d) Percentage and Zebra plots of T_FH_ IL21^+^ cells. (e, f) T_naïve_ and T_FH_ cells were preincubated for 30 min with the inhibitor of STAT3 (Stattic). For STAT3 inhibition, T_naïve_ cells were differentiated to T_FH_, left to rest for 8 h, and then incubated for 30 min with PRL to subsequently determine the MFI of pSTAT3 (histogram). For the plot, pSTAT3 is reported as fold change taken as baseline levels found in T_naïve_ or T_FH_ cells only treated with medium. (f) Percentage and Zebra plots of pSTAT3. Six different experiments were performed; each experiment was done in duplicate. Pooled data are presented as the mean ± SD; ^∗∗∗^*p* < 0.0001 and ^∗^*p* < 0.05 using ANOVA.

To assess whether T_FH_ cells were more active upon PRL treatment, we measured the expression of OX40 and IL21, both molecules serving as activation markers of T_FH_ cells. We found that the T_FH_ cells differentiated in the presence of PRL presented a statistically significant increase in MRL/lpr mice, determined by both expression (MFI) and percentage of OX40 (1359.88 ± 172.05 MFI; 82.85% ± 4.20%), compared with the condition without PRL (1138 ± 76.87 MFI; 70.87% ± 4.07%), likewise, for IL21, with PRL (97.36 ± 4.00MFI;11.90% ± 1.12%) versus without PRL (87.43 ± 1.70MFI;9.27% ± 0.61%). On the other hand, we did not observe any difference in the C57BL/6 mouse cells (Figures 5(a)–5(d)). Moreover, T_FH_ cells derived from MRL/lpr mice expressed more OX40 and IL21 than cells derived from C57BL/6 mice at baseline.

### 3.6. Prolactin Promoted STAT3 Phosphorylation in T_FH_ Cells

It is known that the long PRL isoforms signal through the JAK-STAT and PI3K-AKT pathways [41, 42]. We determined the signaling components associated with the PRL receptor upon activation with recombinant PRL in T_FH_ cells differentiated in vitro. We measured STAT1, STAT3, STAT5, and AKT phosphorylation via flow cytometry. We found that PRL induced phosphorylation of STAT3 (pSTAT3) only in T_FH_ cells derived from MRL/lpr mice and confirmed this PRL activity with an inhibitor of STAT3 (Stattic). The level of pSTAT3 was measured as a fold change (with respect to T_FH_ cells treated with medium) and percentage of positive cells (medium: 1.00-fold change, 8.46% ± 1.00%; PRL: 1.74-fold change, 11.02% ± 1.33%; Stattic: 1.01-fold change, 7.74% ± 1.06%) (Figure 5(e)). In addition, the PRL activity was more prominent in MRL/lpr T_FH_ cells, since the inhibitor significantly reduced pSTAT3 only in the lupus-prone strain. We did not observe pSTAT3 in T_FH_ cells from C57BL/6 mice or in T_naïve_ cells from any mice. In addition, PRL did not induce STAT1, STAT5, and AKT phosphorylation in MRL/lpr mice (Figure S2, Supplementary Materials).

## 4. Discussion

The endocrine system produces hormones that regulate different systems, one of them being the immune system [43]. The bidirectional interactions between the endocrine and immune systems play critical roles in the maintenance of homeostasis. Disturbing mutual communication between these systems might initiate or exacerbate the development of a wide variety of diseases, such as autoimmune thyroid disease [44], rheumatoid arthritis [45], Sjögren syndrome [46], and SLE [47]. Patients with SLE, as well as experimental model mice of the disease (MRL/lpr, NZB/W), show an increase in serum PRL levels associated with the activity of the disease and/or the concentration of IgG autoantibodies [17, 18, 24]. Furthermore, the activity of lupus has also been associated with an increase in T_FH_ cells [36, 48, 49], a subset of helper CD4 T cells that play a crucial role in the generation of antibodies. Indeed, dysfunctional T_FH_ cells can activate autoantibody-producing B cells that cause SLE [50]. Although these studies support that PRL influences T_FH_ cell function in SLE, with a concomitant rise of autoantibodies, the link between PRL and T_FH_ cells is still not clear.

In this study, we present new evidence of the importance of PRL in the development of SLE by increasing the absolute number of T_FH_ cells, the activation of T_FH_ cells, and IL21 secretion in lupus-prone mice. This could favor an uncontrolled response of GCs, faulty tolerance, and an increase in the production of autoantibodies implicated in the pathogenesis of the disease. As it has been observed in the B6.MRL-Faslpr (B6.lpr) and BXD2 strains, the increase in T_FH_ correlates positively with total IgG concentration in serum, as well as with anti-dsDNA antibody levels [51, 52]. We demonstrated here that lupus-prone MRL/lpr mice also presented a positive correlation between the absolute number of T_FH_ cells and the concentration of anti-DNA IgG isotype autoantibodies, as well as a correlation with age. Increased serum PRL levels in these SLE-developing mice are associated with disease exacerbation [24, 53, 54]. Previously, we have also reproduced the exacerbation of the disease by pharmacologically raising serum PRL concentrations with metoclopramide [23, 24]. Here, this same treatment induced an increase in the absolute number of CD4^+^ T cells and T_FH_ cells. Conversely, treating mice with an antagonist of the secretion of PRL (bromocriptine) decreased the absolute number of these cells with respect to mice treated with PBS or without treatment; this behavior was only observed in lupus-prone mice. This increase in T_FH_ cells may be due to an increase in the differentiation of T_FH_ cells, as the expression of BCL6, the master transcription factor of T_FH_ cells [55], was increased only in mice treated with metoclopramide. This increase in the absolute number of T_FH_ cells could give us at least a partial explanation for the association between high levels of PRL and the increase in autoantibodies of the IgG isotype in patients or mice with SLE, as the uncontrolled accumulation of T_FH_ cells might activate autoreactive B cells to produce excessive autoantibodies that cause autoimmune responses [50, 56].

In different reports, it has been shown that PRL is an important factor for both survival and proliferation of different cell types [57, 58]. It has been demonstrated that PRL is an important factor for both the survival and proliferation of early T-cell precursors, such as CD25^+^CD4^−^CD8^−^ double negative cells [10], as well as for the protection of thymocytes from glucocorticoid-induced apoptosis [59]. However, in this work, the prosurvival effects of PRL were not observed in T_FH_ cells differentiated from mature T_naïve_ cells, as happens in the immature B cells of these mice [40]. In addition, there was no effect on the differentiation and proliferation of T_FH_ cells, despite the fact that T_naïve_ and T_FH_ cells expressed the PRL receptor; however, receptor expression was lower in T_naïve_ cells. Furthermore, we did not find evidence of STAT3 activation in T_naïve_ cells, as this kinase was not phosphorylated upon PRL treatment, explaining why the effect of PRL on T_naïve_ cells and their differentiation to T_FH_ was not observed. The increase in the absolute number of T_FH_ cells and the expression of BCL6 (MFI) in vivo may be rather due to an indirect effect of PRL. PRL could be acting on other cells that are helping T_FH_ cells to differentiate. For example, it is known that B cells (follicular and marginal zone) express the PRL receptor and that this expression increases when PRL concentrations rise [24]. On the other hand, it has been reported that IL6 secreted by B cells is important for the differentiation of T_FH_ cells [25]; thus, it will be important to demonstrate, in future tests, if PRL can increase IL6 secretion in B cells, thus favoring the differentiation of T_FH_ cells.

It could also be due to the effect that PRL may have on other hormones that also influence specific components of the immune responses, such as the thyroid-stimulating hormone (TSH). The elevated TSH levels increased the mitogen-induced proliferative response of mouse lymphocytes [60], as well as the percentage of CD4^+^ T cells [61]. Furthermore, serum levels of TSH correlate positively with those of PRL [62], and 11.6% of patients with SLE present elevated levels of TSH [63]. This suggests that in our in vivo tests, the increase in the number of T_FH_ cells in the mice treated with metoclopramide may be due both to an indirect effect of PRL on other cells and to the effect of other hormones such as TSH on CD4^+^ T cells. Therefore, it will be important to study the effect of TSH on T_FH_ cells.

It is probable that the effect of PRL directly occurs in cells that are already differentiated and/or activated where the expression of the receptor is greater. The costimulatory roles of PRL in the in vitro activation of T cells and B cells have been previously reported [5, 64]. In addition, PRL promotes differentiation into CD4^+^ T-bet^+^ T cells [12], CD4^+^ Eomes^+^ T cells [6], and NK cells [8]. T_FH_ cells have a higher expression of the receptor with respect to T_naïve_ cells. This expression increases with age and with the manifestations of the disease in mice that develop SLE, as seen for T cells from patients with SLE, where the T cells express higher levels of the receptor than T cells from healthy subjects [65, 66].

In this work, we demonstrated that T_FH_ cells exclusively express the long isoform of the PRL receptor, finding that PRL could participate in signaling through STAT3 in these cells. An extensive body of evidence links STAT3 with autoimmune diseases. Most of this evidence is related to the capacity of STAT3 to influence the differentiation of lymphoid cells, such as Th17 and Treg CD4^+^ T cells [67]. Stattic has also been used to delay the onset of disease in MRL/lpr mice, reducing the levels of clinical hallmarks of SLE, such as nephritis, renal and skin lesions, proteinuria, and serum autoantibodies [68, 69]. This increase in the phosphorylation of STAT3 when incubating T_FH_ cells with PRL could explain the role of PRL in increasing the percentage (in vitro) and absolute number (in vivo) of IL21-secreting T_FH_ cells (T_FH_ IL21^+^). This is consistent with the observations that the increased IL21 mRNA expression in CD4^+^ T cells from SLE patients is dependent on the activation of STAT3 [70, 71] and that STAT3 directly binds the *IL21* promoter [72, 73]. Furthermore, in mice treated with metoclopramide, the serum levels of IL21 were increased. IL21 serves as a “helper” cytokine to stimulate B cells through interacting with IL21R. IL21 enhances murine B-cell proliferation, IgG class switching, and plasmablast differentiation [74, 75]. Therefore, the increase in IL21 in lupus-prone mice could favor the generation of autoreactive plasma cells and the increase in autoantibodies.

Another effect of PRL on T_FH_ cells was an increase of T_FH_ OX40^+^ cells. OX40 is transiently induced following TCR engagement after antigen (Ag) recognition. Many factors are involved in the kinetics of OX40 expression, including IL21 [76]. IL21 acts in an autocrine way in T_FH_ cells [77]; thus, the PRL-dependent increase in the percentage and number of T_FH_ IL21^+^ cells, as well as the serum levels of IL21, could favor an increase in the percentage of activated T_FH_ cells (OX40^+^). However, it has also been reported that STAT3 plays a direct regulatory role in OX40 mRNA expression in CD4^+^ T cells [78]. Similarly, STAT3 enhances T cell survival by upregulating OX40, BCL2, and Fas ligand [76]. Therefore, the PRL-mediated increase of OX40 on T_FH_ cells could be a direct effect or mediated through IL21. Furthermore, an increased percentage of OX40-expressing CD4^+^ T cells was found in SLE patients, in which it was an indicator of disease activity [79], and the OX40L-OX40 axis was also found to contribute to lupus pathogenesis by promoting the generation of T_FH_ cells [80]. Therefore, PRL influences the immune system in SLE exacerbating the activity of the disease by increasing the number of OX40^+^ T_FH_ cells and activating the OX40-OX40L axis.

## 5. Conclusions

**Figure 6:**
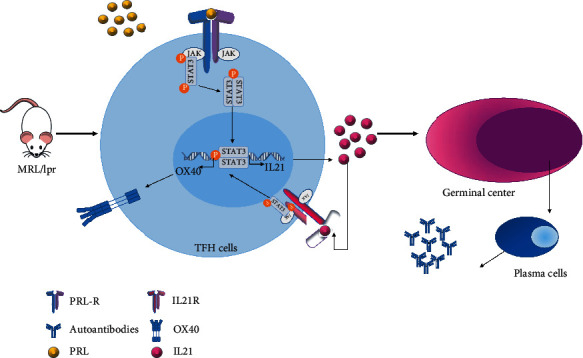
Working model of the mechanism of action of PRL in T_FH_ cells from mice that developed SLE. T_FH_ cells from lupus-prone mice display increased levels of PRL receptor expression restrictive to the long isoform, together with higher levels of serum PRL. This combination results in the heightened activation of STAT3, as well as the increase in OX40 expression and IL21 secretion, which increase the activation of T_FH_ cells favoring the formation of germinal centers, generation of autoreactive plasma cells and increased levels of autoantibodies, with enhanced tissue damage due to the immune complex deposition that characterizes SLE.

Collectively, our data suggest that PRL acts on T_FH_ cells that express the long isoform of the receptor and could participate in signaling through STAT3. We also observed an increase in the number and activation of T_FH_ cells that may favor the formation of GC, interfere with tolerance, and facilitate the generation of autoreactive plasma cells and the secretion of autoantibodies. Therefore, in future studies, it will be important to assess the influence of PRL on the GCs, as well as the interaction of B cells and T_FH_ in an environment featuring high levels of PRL, to better understand the role of PRL in GC formation and to define the most important steps in the pathogenesis of SLE that could be targeted by antagonistic molecules (Figure 6).

## Data Availability

All data included in this study are available upon request by contact with the corresponding author.

## Supplementary Materials

Supplementary Materials Figure S1: absolute number of T_FH_ cells in C57BL/6 mice. Figure S2: PRL does not activate AKT, STAT1, and STAT5 in MRL/lpr mice.
