# Supplementary material for: Prolactin Increases the Frequency of Follicular T Helper Cells with Enhanced IL21 Secretion and OX40 Expression in Lupus-Prone MRL/lpr Mice
Source: J Immunol Res. 2021 Mar 8;2021:6630715. doi: 10.1155/2021/6630715 (PMC7963914; doi:10.1155/2021/6630715)
Supplement: Supplementary Materials — Figure S1: absolute number of TFH cells in C57BL/6 mice. Figure S2: PRL does not activate AKT, STAT1, and STAT5 in MRL/lpr mice. [file 6630715.f1.docx]

Prolactin Increases the Frequency of Follicular T Helper Cells and Their IL21 Secretion and OX40 Expression in Lupus-Prone MRL/lpr Mice

Yolanda P Alemán-García, Ricardo M. Vaquero-García, Rocio Flores-Fernández, Ezequiel M. Fuentes-Pananá, Patricia Gorocica-Rosete, Alberto Pizaña-Venegas, Luis Chávez-Sanchéz, Francico Blanco-Favela, María V. Legorreta-Haquet, and Adriana K. Chávez-Rueda ^1,^*

**Figure S1. Absolute number of T_FH_ cells in C57BL/6 mice**. The 9 week old C57BL/6 mice were treated with metoclopramide (meto, 200 µg/100 µL), bromocriptine (bromo, 0.6 mg/kg), PBS (100 µL), or nothing (age) for 6 weeks. At the end of the treatment, cells were labeled with anti-CD4, anti-CXCR5, anti-PD1, anti-OX40, or anti-IL21 antibodies. The graphs show the absolute number of (a) splenocytes, (b) CD4^+^ T cells, (c) T_FH_ cells, (d) T_FH_ OX40^+^, and (e) T_FH_ IL21^+^. Eight mice were used per condition. Pooled data are presented as the mean ± SD.

**Figure S2.** PRL does not activate AKT, STAT1 and STAT5 in MRL/lpr mice. T_naïve_ cells from 9 week old mice were incubated with PRL and stained to subsequently determine the phosphorylation of (a) pAKT. T_FH_ cells (differentiated in vitro, before resting for 8 h in medium) from 9 week old mice were incubated with PRL and stained to subsequently determine the phosphorylation of (b) pSTAT1, pSTAT5 and pAKT. Six different experiments were performed; each experiment was done in duplicate. Pooled data are presented as the mean ± SD.
